# Effective communication and public engagement strategies to counter misinformation about infectious diseases

**DOI:** 10.1111/imcb.70073

**Published:** 2025-12-12

**Authors:** Sheena Cruickshank, Martin McKee, Christina Pagel

**Affiliations:** ^1^ Lydia Becker Institute of Immunology and Inflammation University of Manchester Manchester UK; ^2^ London School of Hygiene and Tropical Medicine London UK; ^3^ Clinical Operational Research Unit University College London London UK

**Keywords:** Communication, public engagement, public health

## Abstract

Effective communication and public engagement are essential components of infectious disease control, yet they remain underdeveloped in the field of immunology. This review explores how immunologists and scientists can contribute to countering misinformation and improving vaccine uptake through inclusive, culturally sensitive engagement. Drawing on historical and contemporary case studies, we examine how trust, cognitive biases, and community involvement shape public responses. We highlight the importance of co‐produced messaging and the role of community champions in building trust, particularly among marginalized groups. Vaccine communication is analyzed through the lens of the five Cs: confidence, complacency, convenience, communication, and context. We discuss how demographic and structural barriers, historical mistrust, and politicization of health messaging contribute to declining vaccine uptake and propose tailored strategies to address these challenges. The final section focuses on data presentation as a core foundation of public communication, emphasizing that clarity, transparency, and ethical framing are critical to public understanding. We outline principles for designing trustworthy visuals, mitigating cognitive biases, and embedding context directly within graphics to prevent misinterpretation. Participatory approaches to data communication are shown to improve comprehension and trust, especially when co‐developed with affected communities. Together, these domains—engagement, vaccine communication, and data presentation—form a foundation for resilient public health responses. By integrating immunological expertise with inclusive communication strategies, scientists can play a central role in fostering informed decision making and strengthening public cooperation in future outbreaks.

## Graphical abstract

Public trust and cooperation in infectious disease control rest on three pillars: engagement, vaccine communication, and data presentation. Together, these foundations support resilient and inclusive public health responses to misinformation and uncertainty.

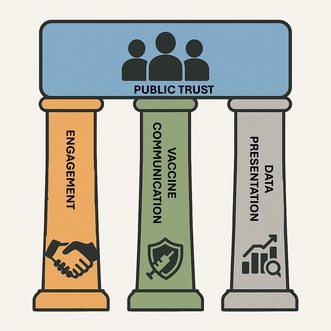

## INTRODUCTION

This review explores the role of immunologists and scientists in public engagement in countering misinformation and improving infectious disease control. Drawing on expertise in public health, vaccine communication, and data analysis and presentation, we examine three interlinked domains: how trust and cognitive biases shape public responses; how culturally sensitive vaccine messaging can enhance uptake; and how data can be communicated most effectively.

Infectious diseases have posed significant threats to public health throughout history, causing widespread illness, economic disruption, and social upheaval. From historical pandemics such as the Black Death to more recent outbreaks of Ebola, COVID‐19, and influenza, controlling and preventing the spread of infectious diseases has remained a major global challenge.^1^ Medical advances, including vaccines, monoclonals, and antiviral treatments, and public health infrastructure, have achieved remarkable successes in disease control over recent decades. That said, public engagement and communication have emerged as critical weaknesses in sustaining the benefits of these efforts. Even the most effective medical interventions can be rendered ineffective without widespread public support. Historical and contemporary outbreaks illustrate this risk. During the 2014–2016 Ebola epidemic, top‐down interventions without cultural sensitivity fueled mistrust and delayed adoption of safe practices. In contrast, New Zealand's COVID‐19 response, which prioritized transparent communication and community engagement, achieved high compliance and low infection rates. These examples, which will be explored later, underscore the importance of effective public engagement.

Kemper *et al*. set out three core reasons to encourage public engagement for public health.^2^ Involving the public in decision‐making is important because it supports democracy– people should have a say in the choices that affect their lives. Second, effective public engagement helps to improve policies and their implementation. When governments listen to feedback from the public, they can make better decisions and build trust. When the public understands the reasons behind and the process of what they are being asked to do, they are more likely to support and accept such requests, especially when they are things that may be controversial. Finally, it ensures that policies reflect what people actually care about as it values everyday experiences and opinions, not just expert advice, and helps avoid decisions that will not work well or that people will reject.

The COVID‐19 pandemic highlighted both the strengths and weaknesses of public engagement in disease control. In some places, decisions were made by those in authority with minimal engagement with those who must implement them or were affected by them. This could easily create public confusion as people struggled to grasp the rationale for what they were being asked to do. It also meant that those making the decisions and those advising them lacked crucial information. An example is the failure, in England, to appreciate the role of agency staff, many working across several facilities, when developing models of COVID‐19 in care homes.^3^ In contrast, some politicians, such as New Zealand's Jacinda Ardern, were able to construct a discourse, in partnership with the media, that portrayed the pandemic response as a collective effort.^4^ The situation was, however, complicated in countries where politicians actively disseminated misinformation and promoted distrust in science, as seen in the differences between states with Republican and Democrat administrations, with corresponding differences in vaccine uptake and mortality.^5^, ^6^

Given this evidence of the importance of public engagement in disease prevention, it is essential to understand the mechanisms that influence public cooperation, the challenges faced in mobilizing communities, and the strategies that can enhance engagement and communication.

In this paper, we examine different strategies used in past and present outbreaks, identifying barriers to effective engagement and communication, and proposing recommendations for improving public health responses. By analyzing case studies and best practices, we will emphasize the importance of a collaborative approach to infectious disease management that builds and maintains trust and prioritizes clear communication, community involvement, and evidence‐based interventions. Ultimately, fostering a well‐informed and engaged public is a vital component of any successful public health strategy.

The paper is structured in three sections: the first examines how public engagement has shaped responses to previous infectious disease outbreaks; the second explores culturally sensitive communication strategies to support vaccine uptake; and the third considers how data presentation influences public understanding and trust.

## UNDERSTANDING PUBLIC ENGAGEMENT IN HEALTH CRISES

### Public engagement strategies for disease prevention and control

Public engagement on disease prevention and control requires deliberate and well‐structured strategies that address all aspects of the public health response. Gilmore and colleagues identify six types of engagement: designing and planning interventions; community entry and trust building; social and behavioral change communication (SBCC); risk communication; surveillance and contact tracing; and broader logistics and administration activities.^7^ They also identified six groups of actors that can be instrumental in this process: community leaders; community and faith‐based organizations; community groups/networks/committees; health management committees; individuals; and key stakeholders, such as survivors and women's representatives. It can take several forms, ranging from consultations to co‐production of policies and interventions^8^ and various types of deliberative democracy, such as citizens' assemblies.^9^

### Case studies of public engagement in disease control

Public engagement has played a crucial role in controlling and preventing infectious diseases throughout history. Examining real‐world case studies provides valuable insights into the effectiveness of different engagement strategies and the challenges in mobilizing public cooperation. This section examines three major public health crises: the COVID‐19 pandemic, the Ebola outbreak, and HIV/AIDS awareness campaigns, highlighting lessons learned from each case.

### COVID‐19 pandemic: Successes and failures in public engagement

The COVID‐19 pandemic, one of the most significant global health crises in recent history, demonstrated both the power and the challenges of public engagement in disease control. Governments and health organizations worldwide implemented a range of strategies to encourage public participation in preventive measures such as social distancing, mask‐wearing, and vaccination. However, their efforts were undermined by widespread misunderstandings of the evidence supporting the measures they were advocating. This was not simply a result of legitimate uncertainty, where some basic information, such as the transmissibility of the virus or efficacy of putative treatments, was unknown in the initial stages of the pandemic. Rather, it was a persistent misunderstanding of some basic concepts, such as viral exponential growth. This is a notoriously difficult concept, as illustrated by the widespread failure of people to incorporate the effects of compound interest in their retirement planning.^10^ Lammers and colleagues conducted a series of experiments early in the pandemic in which they first showed how their sample of Americans tended to make predictions of the course of the epidemic based on implicit assumptions of linear growth, with conservatives more likely to infer a linear model and liberals an exponential one.^11^ However, in subsequent experiments, they showed that explaining exponential growth and guiding subjects through a process to consider the trajectory of growth both improved prediction accuracy and increased support for social distancing. Jäckle and Ettensperger subsequently replicated these studies with politics students in Germany.^12^

Addressing such misunderstandings is facilitated by effective public engagement, whereby politicians and their advisors can better understand how to refine their messages in ways that are most likely to be understood and acted upon. In countries like New Zealand and South Korea, a relatively low spread of the virus has been attributed to strong public engagement. Under Prime Minister Jacinda Ardern, the New Zealand government prioritized transparency, empathy, and clear communication. Frequent press conferences, social media updates, and direct engagement with the public helped build trust. Additionally, strong community‐driven initiatives ensured that individuals followed lockdown measures and supported vulnerable populations. The result was a relatively low number of cases and deaths in the early stages of the pandemic. In Korea, Citizen Forums were used to gather feedback on evolving public health measures, and NGOs helped distribute masks and hygiene kits to underserved communities.

In contrast, other countries struggled with public engagement due to inconsistent messaging, political disagreements, and widespread mis‐ and disinformation (both involve information that is false, but disinformation is created with the intention to deceive).^13^ In the United States, conflicting guidelines from different levels of government, politicization of mask‐wearing, and vaccine hesitancy hindered efforts to control the virus. The rapid spread of misinformation on social media platforms led to public confusion and resistance to safety measures. These challenges underscored the importance of consistent, science‐based communication and the need for proactive efforts to counter mis‐ and disinformation.

In some countries, civil society played a crucial role. The authors of this paper are members of the multidisciplinary group of scientists and practitioners named Independent SAGE. This group delivered weekly online briefings throughout the peak of the pandemic, published numerous reports and statements, and engaged extensively with the media.^14^

The main message from the COVID‐19 pandemic is that public engagement can create a shared understanding of complex issues but must be built on trust, transparency, and culturally sensitive communication. Mis‐ and dis‐information can weaken public cooperation, while clear and empathetic leadership at all levels can enhance it. This is especially important with respect to communities that have traditionally been marginalized and excluded, with growing evidence of how meaningful engagement with community leaders can build and maintain trust.^15^

### Ebola outbreak: Community‐driven approaches to containment

The Ebola outbreak in West Africa (2014–2016) highlighted the critical role of community engagement in controlling a highly lethal infectious disease. Initially, public resistance to health measures posed significant challenges to containment efforts.^16^ Two issues aligned to create misunderstandings that impeded an effective public health response. The first was that many communities viewed foreign health workers with suspicion.^17^ This was understandable given the legacy of colonialism, as Europeans had long been seen as oppressors seeking to exploit the population. However, this was exacerbated by the way that initial public health messages were often delivered in unfamiliar languages and in ways that lacked cultural sensitivity, reinforcing the perception that foreign workers did not understand or respect local customs. Nor was it helped by the appearance of these foreign workers in protective clothing, working in isolation centers where people seemed to go to die, not recover. The second was that these workers had little understanding of traditional burial practices, which involved close physical contact with the deceased, including washing and preparing the body.^18^ As a consequence, communities often rejected guidance, believing that the warnings were exaggerated or part of a foreign agenda.

In response, health officials shifted their approach from imposing top‐down interventions to involving local leaders and trusted community figures. An example was the Social Mobilisation Action Consortium (SMAC) in Sierra Leone.^19^ There were many different forms of community engagement in the affected countries. A study of those employed in Liberia identified treating communities as active participants rather than passive recipients and establishing communication platforms for community engagement ahead of a crisis as good practice.^20^ Those interviewed believed that meaningful engagement improved communication with and increased trust in health authorities and programs, facilitating health system responses and creating a virtuous cycle of increased trust, improved communication, and continued engagement. A review of experiences in the affected countries emphasized the importance of community health workers, working with religious leaders and village elders, to engage with the public and provide information about the virus, promote safe burial practices, and encourage early treatment.^21^

One of the most successful engagement strategies was using survivor advocates, individuals who had recovered from Ebola and could share their experiences firsthand.^22^ These survivors were able to dispel myths, encourage early medical care, and serve as trusted sources of information. However, some caution is required in generalizing this observation as, in some communities, survivors of infections may be stigmatized by fears that they remain contagious, something observed in some places with COVID‐19.^23^ In Liberia, this stigma did, however, decline over time.^24^

The Ebola crisis demonstrated that public engagement is most effective when communities feel a sense of ownership over health interventions. Top‐down approaches often face resistance, but when local leaders and cultural contexts are integrated into the response, engagement improves, and disease control measures are more successful.

### HIV/AIDS awareness campaigns: Long‐term public engagement strategies

The HIV/AIDS epidemic stands as a powerful example of how public engagement, particularly with marginalized communities, can shape the trajectory of a public health crisis. From the earliest days of the epidemic, the gay community played a central role in raising awareness, advocating for research and treatment, and challenging stigma and discrimination.^25^ Their involvement was not only instrumental in improving health outcomes but also in transforming public attitudes and health policy.^26^

In the early 1980s, when AIDS first emerged, the response from many governments was slow and often marked by denial or moral judgment. As soon as it became apparent that men who have sex with men were disproportionately affected, it began to be labeled as a “gay disease,” with those infected facing intense stigma and being frequently blamed for the spread of the disease. This had several consequences. First, politicians were often reluctant to devote resources to a community that lacked widespread sympathy and support. Second, it diverted attention from the other modes of transmission. The challenges faced by those seeking to address these misunderstandings have been well described by Norman Fowler, the Conservative health minister in the United Kingdom (UK) at the time, who struggled to persuade his colleagues, among them Prime Minister Margaret Thatcher, of the need for a large‐scale public campaign that discussed sexual practices that, until relatively recently, had been illegal.^27^

Such efforts were significantly helped by grassroots organizations within the gay community, which became the primary drivers of public engagement in many countries. Groups such as ACT UP (AIDS Coalition to Unleash Power)^28^ and GMHC (Gay Men's Health Crisis)^29^ in the United States, and the Terrence Higgins Trust in the United Kingdom,^30^ mobilized to demand action, educate the public, and support those living with HIV/AIDS.

These organizations pioneered innovative public engagement strategies. They used direct action, public demonstrations, and media campaigns to draw attention to the crisis and pressure governments and pharmaceutical companies to invest in research and make treatments accessible. They also created safe spaces for dialogue, peer support, and education, helping to reduce fear and misinformation. Importantly, they framed HIV/AIDS not just as a medical issue but as a human rights issue, linking health to dignity, equality, and justice.

The gay community's engagement also reshaped the relationship between science and society. Activists demanded inclusion in decision‐making processes, from clinical trial design to policy development. This led to the emergence of community advisory boards and participatory research models, which recognized the value of lived experience alongside scientific expertise. The “Denver Principles” articulated in 1983 emphasized the need for people with AIDS to plan their own care strategies and be involved in all aspects of decision‐making, leading to the establishment of the National Association of People with AIDS to amplify their voices in policy discussions.^31^ The inclusion of affected communities in shaping the response helped ensure that interventions were culturally appropriate, ethically sound, and more likely to be accepted and effective.

Over time, this engagement contributed to major shifts in public health policy. Governments began to fund prevention campaigns, support harm reduction strategies, and expand access to antiretroviral therapy. A pivotal moment came in January 2000, when the United Nations Security Council held its first‐ever discussion on a health issue, recognizing HIV/AIDS as a threat to international peace and security.^32^ This historic meeting led to the adoption of Resolution 1308, which called for coordinated global action and prioritized HIV prevention among peacekeeping forces. By reframing HIV/AIDS as a security concern, the resolution catalyzed political commitment and resource mobilization, particularly in conflict‐affected regions where the epidemic threatened stability. This high‐level engagement demonstrated that sustained public health strategies require not only community involvement but also political leadership at the global level, setting a precedent for integrating health into broader security and development agendas.

Public attitudes also changed, with growing recognition of the need to combat stigma and support those affected. The legacy of this engagement continues today, informing responses to other health crises and reinforcing the importance of involving communities in shaping their own health futures.

The AIDS response demonstrates that public engagement is not just beneficial, it is essential. The leadership of the gay community in confronting HIV/AIDS shows how marginalized groups can drive change when given the space and support to do so. Their efforts helped save lives, change laws, and build a more inclusive public health system. Future responses to infectious diseases must learn from this example, ensuring that those most affected are not only heard but empowered to lead.

### The future of public engagement in infectious disease control

Looking forward, the role of public engagement in controlling and preventing infectious diseases will likely continue to evolve. The rise of digital technologies, the increasing importance of data‐driven decision‐making, and the ongoing challenges of global health disparities all indicate the need for new and adaptive approaches. Understanding how to involve diverse populations, combat mis‐ and disinformation, and use modern tools to encourage preventive behavior will be essential in tackling future outbreaks and maintaining long‐term health improvements.

One promising development in public engagement is the growing use of digital platforms and mobile technology. Throughout the COVID‐19 pandemic, health authorities utilized social media, mobile apps, and online platforms to communicate with the public in real‐time. These tools allowed for the rapid dissemination of information, such as updates on case numbers, vaccination sites, and preventive measures. Moreover, digital platforms can facilitate two‐way communication, where individuals can ask questions, report symptoms, and seek guidance. This shift towards digital communication can make public health messaging more inclusive and accessible, reaching populations who might otherwise have limited access to traditional forms of media. Yet, this is also a double‐edged sword, given the ability of certain platforms to act as echo chambers, increasing polarization.^28^

### Summary

These case studies demonstrate that public engagement is most effective when it is inclusive, culturally sensitive, and community‐led. Whether addressing misunderstandings during COVID‐19, mistrust in Ebola responses, or stigma in HIV/AIDS campaigns, successful strategies have prioritized transparency, local leadership, and co‐production. These lessons are particularly relevant to vaccine communication, where uptake depends not only on access and efficacy but also on trust, context, and the way messages are delivered.

## VACCINATION COMMUNICATION: TRUST, CONTEXT, AND CULTURAL SENSITIVITY

Vaccines are estimated to have prevented the deaths of 154 million children since 2010.^33^ Successful vaccine campaigns led to the eradication of smallpox in 1980. Diseases such as measles were declared eliminated in the United States (in 2000) and the United Kingdom (in 2017). However, vaccines are a victim of their own success– many people have not experienced the severity of measles or polio outbreaks, and vaccine uptake has been steadily declining. This is reflected in countries such as the United States and the United Kingdom losing their measles elimination status. Worryingly, a trend for reduced coverage of childhood vaccines and resurgence of dangerous infections is replicating across the world.^33^ It is not just children who are missing out on important vaccines; data shows that pregnant women,^34^ young adults^35^ and people from a range of demographics such as different ethnic groups^36^ are less likely to take up vaccine offers. For example, lower‐income country, Black or South Asian ethnicity, poverty, language proficiency, religious orthodoxy, belief in science, young age, and educational attainment were all factors that negatively affected vaccination uptake during the COVID‐19 pandemic.^37^, ^38^, ^39^ Infection resurgence is not the only consequence of reduced vaccine uptake. Efforts to eradicate cervical cancer in England by 2040 using the vaccine against human papillomavirus are under threat due to a 17% reduction in vaccine uptake since the COVID‐19 pandemic.^40^ Some healthcare professionals have low levels of vaccine uptake, which is thought to contribute to their reluctance to recommend vaccination.^41^ Nurses and midwives are among the least likely groups of medical workers to be vaccinated.^42^, ^43^ Uptake of vaccines also varies depending on the vaccine type, with the more modern mRNA vaccines raising more concerns than other vaccine types.^44^ Understanding the audience for any campaign and their concerns is essential for effective vaccine communication.

Given these differences, communication must be tailored to each community and context. The five Cs of Confidence, Complacency, Convenience, Communication, and Context are often used to summarize factors that result in reduced vaccine uptake.^45^

Confidence refers to trust in the safety and efficacy of vaccination and can be rooted in mis‐ and dis‐trust. Mistrust describes a general feeling that, for example, health care is unreliable, whereas distrust is more typically rooted in a specific healthcare experience or items of information.^46^ For example, concerns about the rapid development of COVID‐19 vaccines led some individuals to question whether they had been adequately tested.^47^ Historical abuses such as the Tuskegee Syphilis study that deliberately left Black men who had been infected with syphilis untreated have fostered deep‐seated distrust in medical interventions and healthcare institutions among some Black communities.^48^ Such mistrust can be further entrenched if people or their loved ones have a prior negative healthcare experience.^49^ Furthermore, practices by government agencies can further complicate matters. The CIA, in their search for Bin Laden, organized a fake hepatitis B vaccination campaign in Pakistan to obtain DNA from Bin Laden's children, contrary to established ethical principles. This action led the Taliban to ban vaccines in the areas they controlled, as well as broader mistrust in several Muslim communities, with major impacts on efforts to eradicate polio.^50^ Asylum seekers, often under‐vaccinated, may also avoid healthcare due to fear of discrimination and being incarcerated.^51^ Tailored messaging and outreach improves uptake in these groups.^52^

Complacency arises when people underestimate the risk of infection or its consequences. In countries where measles was declared eliminated, many parents came to view the disease as harmless, reducing their motivation to vaccinate their children.^53^ Contemporary misinformation and disinformation can further fuel complacency and vaccine mistrust and distrust. For example, myths about polio vaccines being contaminated with sterilizing agents to target Muslims hindered vaccine uptake.^54^ Politicization of vaccines has become a pressing issue, with, for example, the likelihood of vaccination corresponding with political affiliation in some countries.^55^ Misleading statements by figures in authority or media personalities can feed into parental ideas that “natural” immunity is better and that infections are best controlled by a good diet and lifestyle.^56^, ^57^ In the 2025 outbreak of measles in the United States, it was notable that the Health Secretary Robert F. Kennedy Jr. promoted “natural remedies” such as diet and made statements suggesting the infection was less severe than the vaccine.^58^, ^59^ While he did ultimately acknowledge vaccines were effective, his campaigning most prominently supported natural remedies.^60^

Convenience relates to barriers to access, such as clinic location, opening hours, and cost. In rural areas of low‐income countries, long travel distances and a lack of transport can deter families from attending vaccination appointments.^61^, ^62^, ^63^ Even in high‐income settings, rigid appointment systems or distant vaccine centers can disadvantage shift workers, single parents, or those with limited transport options.^64^ In Scotland, despite pandemic‐related disruptions,^65^ pre‐school immunization rates were maintained or improved.^66^ This success was due to accessible clinic locations, home visits for vulnerable children, and personalized phone reminders.^67^ Flexibility is crucial, especially for low‐income families who may struggle to attend appointments. Vaccine communications must also address digital exclusion^68^ and limited English literacy.

Communication covers the sources and clarity of information as well as who delivers that information. Mixed messages during the COVID‐19 pandemic, such as conflicting or changing advice on mask use by organizations such as WHO,^69^ or the suitability of vaccines for pregnant women^70^ undermined trust and created confusion. Similarly, disinformation on social media, including myths about vaccines causing infertility, persistent tropes impacting several vaccine campaigns^71^ has significantly influenced parental decisions.^71^ Consideration of who is most trusted within defined communities and involving them in communication strategies is also important for enhancing uptake.^72^

Finally, Context encompasses socio‐demographic characteristics, cultural beliefs, and settings. For example, a major challenge in tackling the mPox outbreak of 2022 was stigma due to perceptions about how it was contracted.^73^ This led to a reluctance to be diagnosed that impeded efforts to track infection and carry out effective ring vaccination strategies. Indeed, a systematic review showed a low intention to take up the mPox vaccines by the general public and healthcare workers.^74^ Misunderstanding about whether survivors of Ebola were infectious resulted in people who had recovered suffering severe stigma and social isolation.^75^ The stigmatizing impacts of Ebola were long‐lasting, leading to a reduction in pregnant women seeking healthcare due to fears they would become infected, whether from the hospital or medical staff and concerns about not receiving care.^76^ A lack of awareness of Nigerian culture raised suspicions of aggressive door‐to‐door vaccinators offering free vaccines, with resultant negative impacts on efforts to eradicate polio.^77^ Such experiences emphasize the need for vaccine providers and companies to be sensitive, culturally aware, and politically neutral. Furthermore, campaigns must involve community organizations and leaders in the planning or development of immunization campaigns to avoid groups feeling campaigns are done to them rather than with them.^77^ Effective communication strategies engaged the communities to understand their concerns, address their questions, and co‐develop the public engagement activity. This may include approaches that are most suitable for that community, such as songs, dramatizations, or comics.^78^ Issues around the acceptability of treatments for schistosomiasis in rural Madagascan communities were tackled effectively by co‐developing a song within the communication strategy.^79^

**Figure 1:**
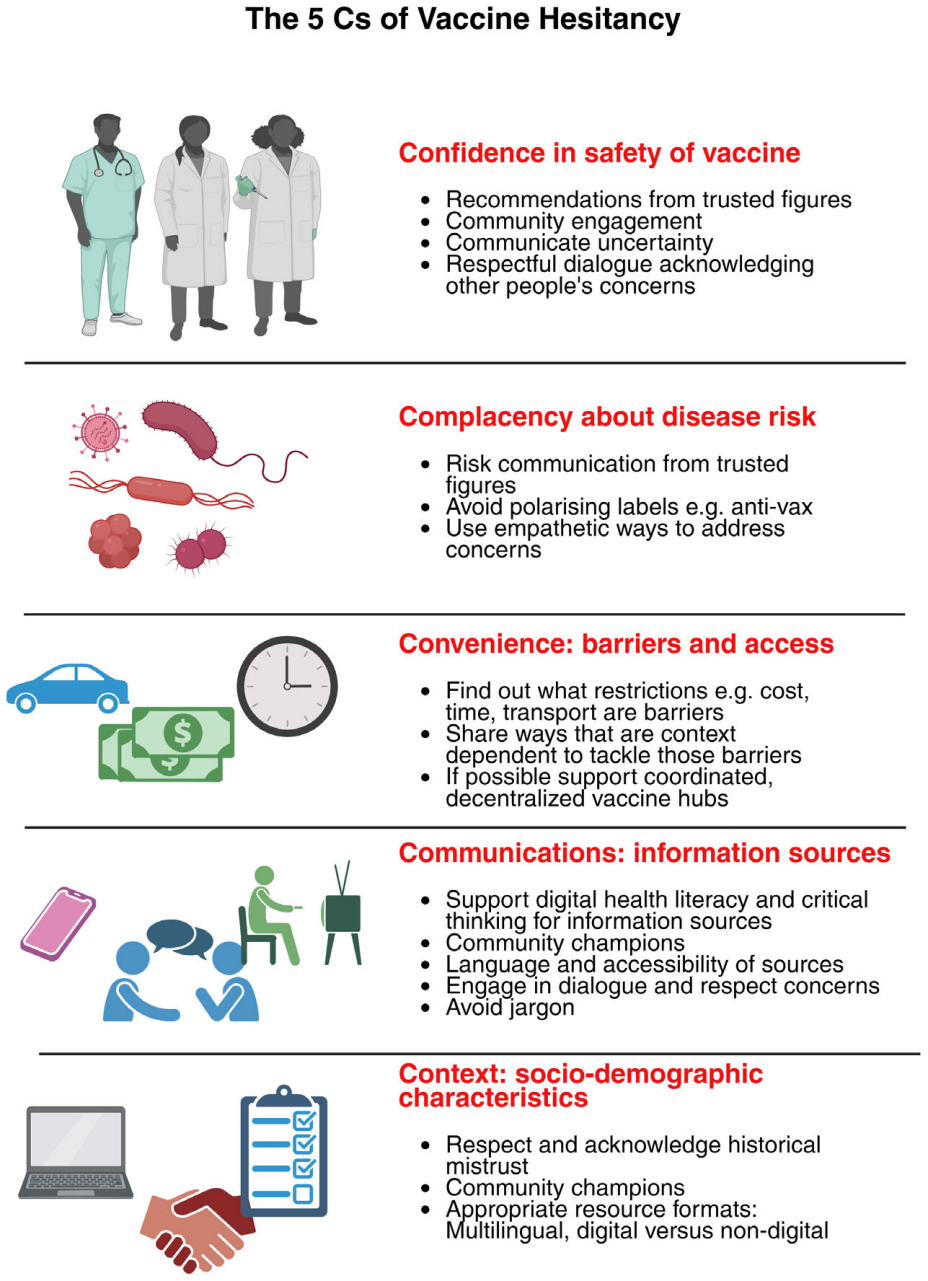
The 5 Cs to tackle declining vaccine uptake: Confidence, Complacency, Convenience, Communications, and Context.

Effective science communication demands understanding all these issues and designing strategies that address them holistically. This means co‐producing messages with communities, taking account of different socio‐demographic characteristics, past experiences dealing with authority, beliefs, and settings (Figure 1). It also means engaging with trusted local voices and ensuring that interventions are culturally sensitive and practically accessible.^80^

### Tackling disinformation and misinformation

Vaccine dis‐ and mis‐information is increasing, especially on social media and even some mainstream media organizations.^81^, ^82^ Care must be taken when addressing them, as raising the profile of anti‐vaccination messages and drawing attention to myths can inadvertently increase concerns, thereby reducing willingness to vaccinate.^83^ Strategies such as scare tactics or only debunking misinformation are also ineffective and, in fact, may further entrench positions. Being open and transparent about vaccine efficacy or risks is more effective than minimizing them. Instead, communicating the weight‐of‐evidence, scientific consensus around vaccines versus related myths, using humor, and incorporating warnings about encountering misinformation is thought to be more effective.^84^ Complementary approaches may aim to enhance digital health literacy and/or help people recognize misinformation online by encouraging critical thinking about the information source, author reliability, and any financial incentives behind the source.^85^ Another valuable strategy involves developing community‐based champions.^72^ This can be among the most effective strategies for vaccine or health advocacy, enabling ‘grassroots’ actors that understand local issues and needs, and helping build trust between leaders, vaccinators, and their communities.^72^

Vaccine‐preventable disease outbreaks pose a growing global risk, and it is vital that we, as immunologists and clinicians, get better at working with communities to tackle uncertainties and enhance uptake. Innovations in vaccine design, such as mRNA cancer vaccines, have the potential to revolutionize health care, including the exciting opportunities of personalized treatments.^86^ However, suspicions coupled with mis‐ and dis‐information about these newer vaccine types risk them being taken up. As new vaccines are constantly being developed against both emerging and existing infectious diseases, and more recently against cancers, it is now of paramount importance to ensure that trust in and acceptance of existing and new vaccines is high.

### Summary

Vaccine uptake is shaped by a complex interplay of trust, cultural context, accessibility, and the way information is communicated. Effective strategies must be tailored to specific communities, address both historical and current sources of mistrust, and avoid reinforcing misinformation through poorly designed messaging. As vaccine technologies evolve and public skepticism persists, the clarity and credibility of data presentation become increasingly important. The following section explores how data can be communicated in ways that support understanding, build trust, and promote informed decision making.

## COMMUNICATING DATA FOR PUBLIC ENGAGEMENT: TRUST, TRANSPARENCY, AND CONTEXT

Effective public engagement requires thoughtful communication; accurate data is not enough, and numbers and research findings do not speak for themselves. The way data are presented and interpreted shapes understanding, behavior, and policy, and different presentations of the same data can lead to people taking away very different messages.^87^ This is compounded by low graphical literacy and limited understanding of statistical concepts among the general public; most people are simply not very good at interpreting charts without training.^88^, ^89^

This section outlines key principles for presenting data in ways that support public understanding, drawing on recent literature and participatory practice.

### Data is not neutral

Data presentation is inherently value‐laden. The framing of data is shaped by institutional priorities and political context.^90^ Data can be used to justify decisions, obscure trade‐offs, or reinforce dominant narratives.^91^ Recognizing this is not a call to abandon data, but to present it with transparency and care. This includes being explicit about what is known, what is uncertain, and what assumptions underpin the analysis.

Once decisions have been made about what data to show, choices about everything from the selection of graph type to the choice of axes, the number of decimal places, and the color schemes can significantly influence interpretation.^92^, ^93^, ^94^, ^95^ For example, the same data on new infections in a population can evoke alarm or complacency depending on whether it is shown as cumulative cases or daily incidence. Data presentation choices are particularly relevant where there is uncertainty, complexity, or contested narratives, such as when communicating vaccine information.

### Cognitive biases and data interpretation

Even well‐presented data can be misinterpreted. Cognitive biases such as confirmation bias, framing effects, and motivated reasoning shape how individuals engage with evidence.^96^ For example, people tend to interpret data in ways that align with their prior beliefs, especially on politicized topics like vaccination.

The World Bank's reflective practice study^97^ found that even highly educated staff misjudged public attitudes due to shared mental models and sunk cost bias. For instance,

42% of Bank staff predicted that ‘most of the poor’ in Nairobi would agree with the statement that “vaccines are risky because they can cause sterilization,” yet in fact only 11% of those poor people who were surveyed in Nairobi did agree with it. Moreover, immunisation rates in that population exceeded 80%. Importantly, proximity to the poor had little effect; they found minimal difference between staff in country offices and those in the Washington headquarters…

Addressing these biases requires not just better data, but better communication.

### Building trust via data presentation

Trust is central to public engagement. Naumova introduced the “The 4E framework‐Evidence, Efficiency, Emphasis, Ethics” to offer a useful guide for designing trustworthy data visuals.^98^

Evidence refers to the credibility and relevance of the data; underlying data should be timely, representative, reliable, and relevant. Efficiency relates to cognitive load and clarity –how easy is it for a reader to interpret the chart appropriately? Emphasis ensures the visual highlights what matters. Make sure that the message you are trying to tell from the data stands out to the reader. Ethics demands accuracy, reliability, integrity, and inclusiveness. Is your data presentation going to mislead or illuminate?

In practice, efficiency and emphasis mean designing visuals that are intuitive, well‐labeled, and sensitive to audience needs. Color use and position should be consistent and unambiguous, so that, for example, higher or lower values are at the same extremes and colors are consistent across multiple charts. Color coding to provide quick interpretation, such as green for better outcomes and red for worse, can be effective but only if the framing is uncontroversial. Otherwise, it risks biasing interpretation. Color choices must also account for accessibility, particularly for viewers with color vision deficiency. Axes should almost always start at zero if zero is a natural lower limit, and vertical axis scales should be kept constant across series of charts to avoid giving false impressions of variability and to allow for quick visual comparison across charts.

Avoid overloading visuals with information. Most viewers do not read axes carefully, and many are unfamiliar with chart conventions. Layering information in presentations, online graphics, or even written materials, such as using progressive reveals, annotations, or tooltips, can help manage cognitive load. Certain common data formats, such as logarithmic axes, 3D charts, or complicated quantities (such as ratios or derived variables), require explicit explanation and are still often misinterpreted. Where such formats are necessary, explanatory text on the chart itself or interactive walkthroughs can improve understanding.

Precision should be used judiciously; Does the most precise version of a quantity aid understanding? Does it provide a false impression of accuracy? In many cases, rounding to three, or even two, significant figures improves clarity. For example, people find it easier to compare “2500” and “6200” compared to “2469” and “6243.” Decimal places often obscure rather than help, so only include them if precision to that level is necessary for interpretation. In addition, if there is significant uncertainty around numerical quantities, rounding can signal appropriate caution. Over‐precision can imply false certainty, and if key quantities (e.g., incidence of adverse vaccine reactions) are later revised, it can damage public trust even if the overall message (e.g., that adverse reactions are rare) remains the same.

For key concepts, it is often helpful to explain the same idea in multiple ways, to allow for different cognitive preferences in the audience. For example: “reduced by 50%” and “about half as much,” or “if before 8 out of 100 people would get sick, now it's only 4 out of 100.”

It is essential to present both relative and absolute numbers when discussing efficacy or changes in risk. For instance, a “100% increase in risk” sounds alarming, but if the baseline risk is 0.001% (1 in 100 000), the practical impact of moving to a risk of 2 in 100 000 is minimal. Providing both perspectives helps audiences assess the relevance to their own lives.^99^, ^100^, ^101^

Avoid jargon wherever possible, being mindful that words that seem everyday and ordinary to you are nonetheless jargon.^102^, ^103^ The best way to assess jargon is not by relying on you or your peers. Instead, try running intended language and explanations by interested non‐experts. Always consider what background knowledge the audience needs to interpret the data. This will differ by context. For some audiences, it may be appropriate to use approximate explanations, for instance, talking about “immune cells” rather than distinguishing between T and B cells, if the distinction is not central to the message you are trying to convey. Clarity should take precedence over technical completeness.

In digital environments, particularly social media and blogs, charts are frequently shared without accompanying text, or shared in such a way that the chart can be copied without the text to place it in context (e.g., an image included in a tweet). To prevent misinterpretation of screenshots or reposts, key context must be embedded directly within the visual itself. This includes specifying the data source, time frame, population, any caveats or assumptions, and key takeaway message *within* the graphic. Without this, charts risk being detached from their intended meaning and repurposed to support misleading narratives. Stokes *et al*. have shown that readers actively prefer more text to help them interpret graphical data.^104^

Trust in scientists and public health agencies strongly predicts vaccine uptake.^105^ Yet, institutional trust is unevenly distributed. Scientific voices explicitly positioned as independent can play a vital role in bridging gaps between official data and public concerns.^14^

### Narrative visualization and data storytelling

Narrative visualizations combine the clarity of static graphics with the explanatory depth of simulations.^106^ Narrative visualizations guide the viewer through a data story, showing not just outcomes but mechanisms. This is particularly useful for communicating difficult or counterintuitive concepts such as cumulative risk, exponential growth, or complex trade‐offs.

For example, a narrative visualization of COVID‐19 transmission at a family gathering was more effective than a static chart or anecdote in increasing concern and perceived risk, through increased understanding of the mechanism.^106^ This suggests that narrative formats can help overcome skepticism and disengagement. In immunology, narrative visuals could show how immunity builds over time, how different disease variants affect transmission, or how community‐level protection emerges. They can also highlight the human stories behind the data, making abstract numbers more relatable.

### Participatory approaches and data Co‐production

Clear presentation of data is enhanced through co‐production with intended audiences. Co‐production involves working with communities on how best to support the interpretation and understanding of scientific concepts or essential data.

During COVID‐19, participatory approaches were underutilized, leading to “monothink” and missed opportunities for inclusive communication.^97^ In contrast, author Pagel led a co‐production project with families of children with congenital heart disease to design a graphical representation of national audit results. The statistical team all reported how this process highlighted possible misinterpretations–and mitigations–of data visuals that would never have occurred to the team without that parent voice.^107^, ^108^ Co‐produced data stories can also challenge dominant narratives. For example, community‐based vaccine champions have been effective in countering misinformation and building trust.^105^ These approaches require investment in facilitation, translation, and feedback mechanisms, but they offer a powerful complement to top‐down messaging driven by topic experts.

If time or resources are too scarce to allow a complete co‐production process in designing visuals, a rapid “red team” process for charts can still be beneficial. This involves asking a few others, who have not seen the charts before and are (preferably) not topic experts, to explain the key messages conveyed by the charts. This can be extremely useful in picking up areas where data presentation can be rapidly improved.

### Summary

Data is essential to immunology, but it must be communicated with care. In an era of misinformation, politicization, and distrust, the challenge is not just to present data, but to engage people with it. This requires attention to design, context, narrative, and participation. By embedding data communication within the 5Cs framework and drawing on ethical, cognitive, and participatory principles, we can make data not just visible, but meaningful.

## CONCLUSION

Across global and historical responses to infectious disease outbreaks, effective public engagement has consistently relied on trust, cultural sensitivity, and inclusive communication. Vaccine uptake, in particular, is shaped by these factors, requiring tailored strategies that address historical mistrust, misinformation, and structural barriers. Underpinning both engagement and vaccine communication is the way data are presented. Clarity, transparency, and ethical framing are essential to support understanding and informed decision‐making. Together, the three domains of engagement, vaccine communication, and data presentation form a foundation for resilient public health strategies that are responsive to diverse communities and grounded in evidence.

## CONFLICT OF INTEREST

We declare that we have no conflicts of interest.

## Data Availability

Data sharing not applicable to this article as no data sets were generated or analyzed during the current study.
